# Estimating the prevalence alcohol use disorder and relapse risks in Kermanshah Province of Iran: Integrating direct, indirect methods, and tree-based analysis

**DOI:** 10.1371/journal.pone.0325107

**Published:** 2025-08-18

**Authors:** Faezeh Tatari, Mostafa Alikhani, Farnaz Radmehr, Sara Hookari, Mehran Kamani, Vahid Farnia, Omran Davarinejad, Mehdi Moradinazar, Lida Olfati, Narges Nazari Harmooshi, Safora Salemi, Bahareh Rahami

**Affiliations:** 1 Department of Psychiatry, Substance Abuse Prevention Research Center, Health Institute, Kermanshah University of Medical Sciences, Kermanshah, Iran; 2 Clinical Research Development Center, Imam Khomeini and Mohammad Kermanshahi and Farabi Hospitals, Kermanshah University of Medical Sciences, Kermanshah, Iran; 3 Sirjan University of Medical Sciences, Sirjan, Iran; 4 Department of Psychiatry, School of Clinical Sciences, Faculty of Medicine, Nursing and Health Sciences, Monash University, Clayton, Victoria, Australia; 5 Infectious Diseases Research Center, Kermanshah University of Medical Sciences, Kermanshah, Iran; 6 Health in Emergency and Disaster Research Center, University of Social Welfare and Rehabilitation Sciences, Tehran, Iran; National University of Sciences and Technology, PAKISTAN

## Abstract

**Background:**

Alcohol Use Disorder (AUD) is a major health issue in various societies, particularly in Iran, with detrimental consequences. In this study, we examined the prevalence of AUD using direct and indirect methods (Network Scale-Up model (NUS) and Crosswise model (CM)), as well as the factors associated with relapse, utilizing tree analysis.

**Methods:**

In this cross-sectional study conducted among individuals aged 15–69 in Kermanshah Province, western Iran, 6,127 participants were selected using stratified sampling. Data collection was performed electronically and recorded in a digital system by trained interviewers. The indirect assessment utilized both NUS and CM methods and Factors affecting the recurrence of alcohol use were investigated using the tree regression method.

**Results:**

The prevalence of AUD in western Iran was estimated at 3.20% using direct methods, 20.54% using the CM method, and 7.21% using the NUS method. A total of 34 (0.7%) individuals reported relapse in AUD. Tree analysis results indicated that the frequency of relapse (chi square = 4994.0, P < 0.001) and a history of physical illness in the family (chi square = 6.0, P = 0.031) were influential factors in alcohol relapse.

**Conclusion:**

The findings of this study provide insights into the actual levels of AUD and factors associated with relapse, enabling healthcare providers to make better decisions for preventive planning to reduce AUD and improve treatment outcomes.

## Introduction

Alcohol is a psychoactive substance with dependence-producing properties. Alcohol Use Disorder (AUD) and problems related to alcohol vary widely around the world, but the burden of alcohol-related disease and death remains significant in most countries [1,2]. Harmful use of alcohol ranks among the top five risk factors for disease, disability, and death throughout the world [3].

In a study, the overall prevalence of AUD in Iran was reported to be 12.0% for the general population and 15.0% for youth. The prevalence of AUD varies from 0.03% to 68.0% in different regions, 0.3% to 66.6% among men, and 0.2% to 21.0% among women [4]. According to the World Health Organization, the lowest per capita AUD is observed in countries with a predominantly Muslim population, including Iran. Although the overall prevalence of AUD in these countries is low, it has increased in recent years due to various reasons, such as the availability of alcohol in stores, changes in social-cultural norms, and shifting beliefs [5]. In fact, individuals who consume alcohol in Muslim countries may engage in binge drinking and consume larger quantities of alcohol [6].

At the same time, it should be noted that the starting concentration of alcohol consumption among Iranian youth is high; this concentration is 3% in Europe and 40% in our country. Regardless, the issue of alcohol addiction is undeniable and, unfortunately, alcohol consumption is a precursor to many social harms [7]. Cancers of the mouth, throat, larynx [8], esophagus, liver, and breast are all associated with alcohol consumption. Alcohol may also increase the risk of colon and rectal cancer [9]. Therefore, combating alcohol consumption should be prioritized by health organizations and considered a multi-sectoral program in society [10].

The production, sale, and consumption of alcohol are illegal in Iran. However, the use of homemade alcoholic beverages or smuggled drinks from abroad is common [11]. A growing body of evidence regarding alcohol consumption in Iran is emerging, with numerous studies reporting on anxiety disorders, aggressive behaviors, and alcohol-related harms [12,13].

One common problem among individuals with AUD is relapse after undergoing withdrawal. Relapse is a multifactorial phenomenon influenced by individual patient characteristics, the substance used, and environmental reinforcers [14]. Although there are many definitions of relapse, it is generally considered a return to previous patterns of substance use [15].

Among the significant public health challenges is the inability to accurately measure hidden or inaccessible populations with risky behaviors. Given the lack of accurate statistics on AUD in Kermanshah Province and the need for epidemiological information as a basis for preventive planning, treatment, and reduction of its consequences, this information is essential. Considering the relatively high prevalence of AUD and its harmful effects in causing various diseases and increasing mortality rates, preventing and examining the factors related to relapse is an important task for health professionals. If relapse occurs, the likelihood of premature death increases, and reducing the associated treatment costs is also crucial. Although there are numerous reports on relapse among alcohol users, none have utilized decision trees (DT). DT is a straightforward tool for health policymakers and healthcare providers. This research, conducted in Kermanshah Province, investigates the prevalence of alcohol consumption using direct and indirect methods and examines the factors related to their recurrence using tree analysis, aiming to inform targeted preventive planning at the primary health care level and reduce relapse.

## Methods

### Study design

The present study was a cross-sectional study conducted to investigate the prevalence of Alcohol Use Disorder (AUD) directly and indirectly as well as the factors related to relapse of AUD among people aged 15–69 years in Kermanshah Province, western Iran. The start date of the study was 02.05.2019 and the end date was 02.08.2020.

### Sampling and sample size estimation

The statistical population of the study included the entire population aged 15–69 years across the whole province (1,952,434 people). The sampling method was stratified in proportion to the total sampling fraction, among cities and then systematically within cities. The total population of Kermanshah Province was determined based on 14 cities of this province with the table of population reports of the provinces determined by age groups (based on the 2016 census). With 95% confidence interval and 3% accuracy, the required sample size according to the type of study was n = 6127.

The study inclusion criteria included both genders (male and female), age range of 15–69 years, and being native of Kermanshah Province (residence at least 5 years in the province). The study exclusion criteria included mental illnesses, physical disability, unwillingness to participate, and lack of collaboration in this study.

### Data collection

For data collection, a direct method and two indirect methods were used:

#### 1. Direct method.

To assess whether or not the subjects had a history of AUD a question was asked: “Have you used alcohol more than one time during a lifetime question were “Yes” or “No”.

#### 2. Crosswise method (CM).

The CM relies on a simple design that only requires respondents to provide one simple yes/no-answer to a set of two different questions without burdening them with complex instructions or activities [16]. While one question directly asks about the sensitive attitude (alcohol consumption), the other one directly enquires about an unrelated non-sensitive topic. However, respondents are instructed to provide only one answer to both questions. Response A, if the answers to both questions are the same (either both ‘yes’ or both ‘no’); Response B, if the answers to the two questions differ (either ‘yes’ and ‘no’ or ‘no’ and ‘yes’). Sample CM question (Are you born in winter?/ Have you used alcohol in the past 6 months?)

#### 3. Network scale-up method (NSU).

The NSU method estimates population sizes using information about the personal networks of survey respondents under the assumption that personal networks are, on average, representative of the general population [17]. For example, if a respondent report knowing 2 alcohol users and knows 200 people overall, we can estimate that 2/200, or 1%, of the population are alcohol users. This estimate can be improved by averaging data over many respondents [18].

Based on the study of Rastgari et al [19], in this study, the size of the social network was considered n = 308, which means that each person over the age of 15 years would know 308 people. The individuals introduced by the interviewee should have had a phone call, face-to-face contact, or e-mail with them at least once in the past year; they should know each other by name and face, and they know them and, whenever necessary, they should be able to easily contact them [20]. The data needed for the network scale-up method come from interviews with a random sample of the general population. Sample NSU method question (Among your relatives and neighbors, friends and colleagues who live in your city, how many people do you know who use alcohol?).

### Data management

In 17.10.2019, the data was used for research purposes. The data were collected electronically and recorded in a digital system. Initially, the surveyors, who were all psychologists, introduced themselves by their ID card, presented the research plan and explained its objectives, which included awareness of the current situation and knowledge of the current state of alcohol consumption for targeted preventive planning at the level of primary health care and finally to reduce alcohol consumption among the people. Regarding, the information of individuals remained confidential and none of their information was used instrumentally, and given that the information was collected without anonymity, all contributing to their satisfaction and confidence to participate in the study.

### Data analysis

To identify factors influencing AUD relapse in individuals and predict the dependent variable of AUD relapse based on independent demographic variables including age, marital status, education level, occupation, residential area, economic conditions, personal history of mental illness, personal history of physical illness, family history of mental illness, family history of physical illness, family history of addiction, and alcohol consumption history including duration of alcohol use, frequency of alcohol use per week, family history of alcohol use, history of alcohol withdrawal, duration of Alcohol withdrawal, history of alcohol relapse, Frequency of AUD relapse, and reasons for AUD relapse, individuals were classified into two levels of AUD relapse history (Has history of AUD relapse, Does not have history of AUD Relapse) from the tree regression method with the method Chi-squared Automatic Interaction Detection (CHAID) was used.

Here, before implementing the regression tree method, the assumptions of this method were examined. Additionally, the dependent variable of alcohol relapse history was categorized into two levels with nominal scale, defined as having a history of alcohol relapse and not having a history of alcohol relapse, and all these levels were included as target classes in the study. The maximum number of growth levels for the tree was set to 3 by default. Due to sample size limitations, the minimum number of cases in the parent node was set to 20 and in the child node to 10. Nodes that did not meet this criterion were excluded from the tree divisions. The significance level for differentiating nodes or merging classes was set at 0.05. The risk value was determined based on standard errors, set to a positive number equal to 1. In the CHAID tree growth method, missing values were treated as a floating class and allowed to merge with other classes in the tree nodes.

### Measures

We assessed gender (male and female); age (years); marital status (single, married vs divorced and other); Educational level (people with lower literacy level, Primary vs high school, diploma and High diploma ≥); Occupation (student, self-employment, employed, unemployed, housewife and retired); Residential areas (urban and rural); Economic status [21] (excellent, good, middle, low and N/A); history of mental illness (at lifetime) (Mental illnesses based on self-reported information and history of hospitalization and referral to a psychologist and psychiatrist (yes, no and N/A)); history of physical illness (physical illness based on self-reported information and history of hospitalization and referral to physician (yes, no and N/A); family history of mental illness (yes and no); history of chronic physical illness in the family (yes, no and N/A); family history of SUDs (Diagnosis of SUDs based on diagnostic and statistical manual of mental disorders, fifth edition (DSM-5) and as ascertained by trained and experienced psychologists (yes, no and N/A)); Duration of alcohol consumption(years); Frequency of alcohol consumption per week (number); History of alcohol consumption in the family(yes, no); History of abstinence from alcohol consumption(yes, no); Duration of abstinence from alcohol consumption (years); History of alcohol relapse (yes, no); Frequency of relapse of alcohol consumption (number)

### Ethical considerations

All participants gave their signed written informed consent. Furthermore, informed written consent was obtained from all parent and/or legal guardian of the participants who were below 16 years of age. The Medical Research and Ethical Committee of National Institute for Medical Research Development (Iran; registration No. IR.NIMAD.REC.1398.031) approved the study, which was performed under the ethical principles laid down in the seventh and current edition (2013) of the Declaration of Helsinki.

## Results

Among the participants in this study, which was conducted in Kermanshah Province, 33293 participants were male (53.70%), and 2454 participants (40.05%) were in the age group of 31–45 years. For marital status, 3807 participants (62.13%) were married. Also, 1688 participants (27.55%) had primary and high school education, and 1607 participants (26.23%) had diploma education. For employment status, 2377 participants (38.80%) were self-employed and 647 participants (10.56%) were unemployed, with 5938 participants (96.93%) living in the city. The economic status, 3818 participants (62.31%) had a moderate status and 731 participants (11.93%) had a low status. Further, 271 participants (4.42%) had a history of mental disease, 797 participants (13.01%) had a history of physical diseases, 228 participants (3.73%) had a family history of mental diseases, 962 participants (15.70%) had a history of chronic physical diseases in the family, and 638 participants (10.41%) had a family history of SUDs. Other demographic information is presented in Table 1.

**Table 1:** Demographic, clinical characteristic and history of alcohol consumption of the participant.

Variable	Levels	Total n (%)	Male n (%)	Female n (%)	P value
Age (years)	15-30	2152 (35.12)	989 (30.02)	1163 (41.05)	86.88 (<0.0001)
	31-45	2454 (40.05)	1407 (42.71)	1047 (36.96)	
	46-60	1185 (19.34)	683 (20.73)	502 (17.72)	
	61≥	336 (5.48)	215 (6.53)	121 (4.27)	
Marital status	single	1938 (31.63)	1049 (31.85)	889 (31.38)	85.30 (<0.0001)
	married	3807 (62.13)	2126 (64.54)	1681 (59.34)	
	divorced and other	382 (6.23)	119 (3.61)	263 (9.28)	
Educational level	people with lower literacy level	502 (8.19)	193 (5.86)	309 (10.91)	88.381 (<0.0001)
	Primary and high school	1688 (27.55)	1007 (30.57)	681 (24.04)	
	diploma	1607 (26.23)	882 (26.78)	725 (25.59)	
	High diploma ≥	2330 (38.03)	1212 (36.79)	1118 (39.46)	
Occupation	student	666 (10.87)	292 (8.86)	374 (13.20)	2694.56 (<0.0001)
	self-employment	2377 (38.80)	2032 (61.69)	345 (12.18)	
	employed	920 (15.02)	585 (17.76)	335 (11.85)	
	unemployed	647 (10.56)	334 (10.14)	313 (11.05)	
	housewife	1473 (24.04)	14 (0.43)	1459 (51.50)	
	Retired	44 (0.72)	37 (1.12)	7 (0.25)	
Residential areas	urban	5938 (96.93)	3208 (97.37)	2731 (96.40)	5.015 (0.015)
	rural	188 (3.07)	86 (2.61)	102 (3.60)	
Economic status	excellent	261 (4.26)	138 (4.19)	123 (4.34)	3.18 (0.528)
	good	1295 (21.14)	707 (21.46)	588 (20.76)	
	middle	3818 (62.31)	2032 (61.69)	1786 (63.04)	
	low	731 (11.93)	402 (12.20)	329 (11.61)	
	N/A	22 (0.36)	15 (0.46)	7 (0.25)	
History of mental illness	yes	271 (4.42)	110 (3.34)	161 (5.68)	22.67 (<0.0001)
	no	5834 (95.22)	3176 (96.42)	2658 (93.82)	
	N/A	22 (0.36)	8 (0.24)	14 (0.49)	
History of physical illness	yes	797 (13.01)	386 (11.72)	411 (14.51)	10.529 (<0.005)
	no	5309 (86.65)	2896 (87.92)	2413 (85.17)	
	N/A	21 (0.34)	12 (0.36)	9 (0.32)	
Family history of mental illness	yes	228 (3.73)	106 (3.23)	122 (4.33)	6.568 (0.037)
	no	5877 (96.27)	3179 (96.77)	2698 (95.67)	
History of chronic physical illness in the family	yes	962 (15.70)	409 (12.42)	553 (19.52)	58.065 (<0.0001)
	no	5138 (83.86)	2870 (87.13)	2268 (80.06)	
	N/A	27 (0.44)	15 (0.46)	12 (0.42)	
Family history of SUDs	yes	638 (10.41)	303 (9.20)	335 (11.82)	12.340 (0.002)
	no	5448 (88.92)	2971 (90.22)	2476 (87.40)	
	N/A	41 (0.67)	19 (0.58)	22 (0.78)	
Duration of alcohol consumption	0-5	16 (8.7)	16 (9.8)	0 (0.0)	4.85 (0.028)
	6-10	7 (3.8)	5 (3.0)	2 (10.5)	
	11-15	4 (2.2)	4 (2.4)	0 (0.0)	
	>15	156 (85.2)	139 (84.8)	17 (89.5)	
Frequency of alcohol consumption per week	1	144 (75.4)	123 (72.4)	21 (100.0)	7.701 (0.005)
	2	35 (18.3)	35 (20.6)	0 (0.0)	
	3	9 (4.7)	9 (5.3)	0 (0.0)	
	4	3 (1.6)	3 (1.8)	0 (0.0)	
History of AUD in the family	yes	373 (6.1)	187 (5.7)	186 (6.6)	2.22 (0.136)
	no	5700 (93.9)	3084 (94.3)	2616 (93.4)	
History of abstinence from alcohol consumption	yes	27 (0.5)	23 (0.8)	4 (0.2)	9.15 (0.002)
	no	5376 (99.5)	3024 (99.2)	2352 (99.8)	
Duration of abstinence from alcohol consumption	0-5	14 (58.3)	13 (61.9)	1 (33.3)	2.45 (0.118)
	6-10	3 (12.5)	3 (14.3)	0 (0.0)	
	>15	7 (29.2)	5 (23.8)	2 (66.7)	
History of alcohol relapse	yes	34 (0.7)	28 (1.0)	6 (0.3)	8.897 (0.0029)
	no	5113 (99.3)	2912 (99.0)	2201 (99.7)	
Frequency of relapse of AUD	1	7 (21.2)	7 (25.0)	0 (0.0)	11.16 (0.0008)
	2	13 (39.4)	10 (35.7)	3 (60.0)	
	3	10 (30.3)	10 (35.7)	0 (0.0)	
	4	2 (6.1)	1 (20.0)	1 (20.0)	
	5>	1 (3.0)	0 (0.0)	1 (20.0)	

**Fig 1:**
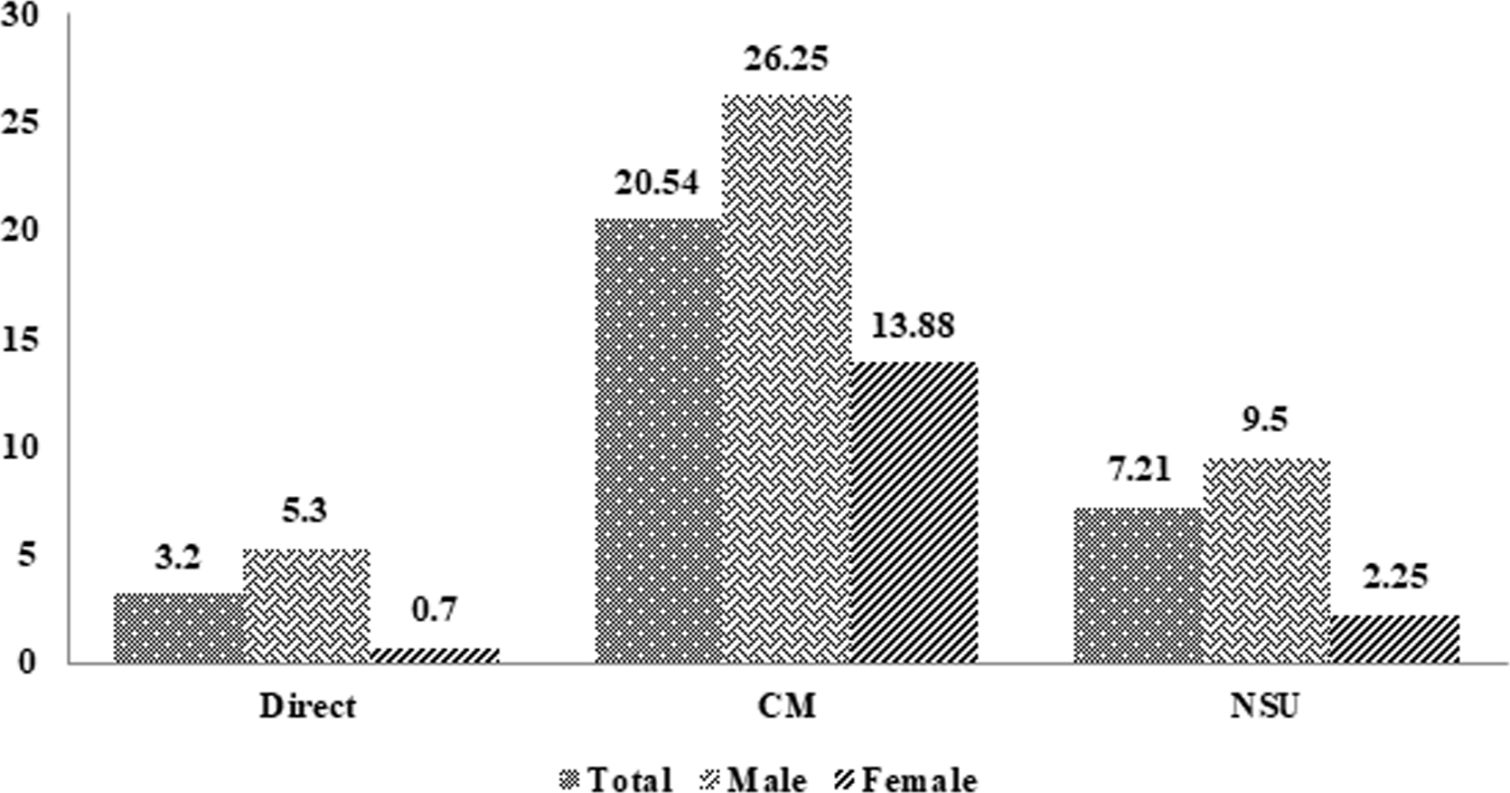
Prevalence of Alcohol Use Disorder by direct and indirect (NUS and CM) methods in Kermanshah province.

The results showed that the prevalence of AUD using direct methods in Kermanshah Province was 3.20%, with a prevalence of 5.30% in men and 0.7% in women. The results indicated that the prevalence of AUD using the indirect CM method in Kermanshah Province was 20.54% (26.25% in men and 13.88% in women). According to the indirect NUS method, the prevalence of AUD in Kermanshah Province was reported at 7.21% (9.50% in men and 2.25% in women) (Fig 1).

A total of 34 (0.7%) individuals reported AUD relapse. Among with AUD, over 80% (156 individuals, 85.2%) reported using alcohol for more than 15 years. The majority of individuals (144, 75.4%) reported their frequency of alcohol use per week as once. Additionally, the frequency of AUD was reported to be significantly higher in men than in women. A total of 373 (6.1%) individuals had a family history of AUD, and 14 (58.3%) individuals had not consumed alcohol for less than 5 years. Over 90% of individuals reported relapsing into alcohol use at least 3 times after abstinence (Table 1).

**Fig 2:**
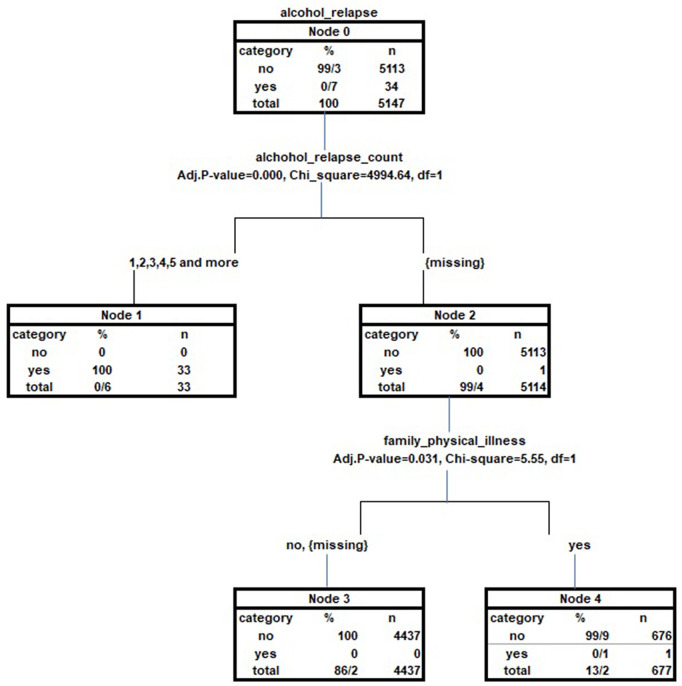
Classification diagram of history of relapse of AUD according to the number of relapses of AUD and history of physical illness in the family based on the decision tree.

Based on the tree diagram resulting from the regression tree analysis, the factor of the frequency of AUD relapse (p < 0.001) was identified as the best predictor of the dependent variable of AUD relapse history. Subsequently, the family history of physical illness (p < 0.05) also showed a statistically significant impact in explaining the history of AUD relapse and was included in our regression tree model. In the root node (Node 0), a total of 5,147 individuals with varying levels of AUD relapse history were included in the study, of which only 34 (0.7%) had a positive history of AUD relapse. In the first level of the tree, individuals with one to more than five instances of AUD relapse history were merged into the first group (Node 1) due to the lack of statistically significant differences in the dependent variable of AUD relapse history. For Those who with indeterminate frequencies of alcohol relapse that were placed in a second group (Node 2), the family history of physical illness became the only significant predictor of alcohol relapse.. Thus, at the second level of the tree model, in Nodes 3 and 4, individuals who reported a positive or negative family history of physical illness had their AUD relapse history classified into two categories: “Positive” and “Negative.” In Node 3, all individuals without a history of AUD relapse were reported, while Node 4 contained 99.9% of individuals with a history of alcohol relapse, representing the majority of that category.. The risk estimate, which is a measure of the predictive accuracy of the decision tree, reflects the ratio of cases incorrectly classified into other categories of the dependent variable of AUD relapse history. The correct classification rate for patients with a positive AUD relapse history was approximately 97.1%, while for patients with a negative alcohol relapse history, it was 100%. Here, the risk value (0.001) and the classification table indicated that the overall correct classification rate in our tree model was nearly 99.9% (Fig 2). It is worth noting that other predictor variables included in the regression model were excluded from the tree model due to the lack of significant statistical interaction with other factors present in predicting AUD relapse history.

## Discussion

The present study was conducted to investigate the prevalence of Alcohol Use Disorder (AUD) and to estimate its prevalence using two direct and indirect methods (NUS and CM) as well as to determine the main factors related to relapse in Kermanshah Province (western Iran). According to the study results, it was found that the prevalence of AUD in Kermanshah Province was estimated 3.20% by direct methods, 20.54% by indirect CM method and 7.21% by indirect NUS method. The rate of AUD in male was nearly seven times that of female.

Our findings indicate that the per capita alcohol consumption in Kermanshah is lower than the average alcohol consumption in Iran, which is reported to be 13% according to the study by Chaghin and et al. [4]. This rate is also significantly lower compared to global statistics, which is an expected result. A study on global alcohol consumption estimated that the rate of alcohol consumption decreased from 46% in 1990 to 43% in 2017, with predictions suggesting it will reach 40% by 2030 [22]. This study also states that the per capita alcohol consumption in the Middle East and North African countries is the lowest compared to other countries, which may be due to the fact that most residents in these areas are Muslim, and abstaining from alcohol is part of their religious teachings [22]. In fact, the lower alcohol consumption in Iran may stem from the prevailing conservative socio-cultural and religious context and the criminalization of alcohol consumption by the Islamic government in charge. These findings suggest that Iran’s approach to alcohol control policies should go beyond a heavy reliance on the criminalization of alcohol consumption and adopt a culturally sensitive evidence-based public health approach, where comprehensive primary prevention strategies (such as developing educational programs for the public and school-based prevention programs) are more widely implemented [11].

Our findings may be related to the prevailing socio-cultural practices and gender norms in conservative and patriarchal contexts like Iran, where the level of tolerance and acceptance of alcohol consumption is higher for male compared to female [23–25]. It is expected that AUD estimates in Iran are likely underestimated due to the stigma and illegality surrounding alcohol use, which may encourage individuals to hide or underreport their alcohol consumption in direct face-to-face studies. In fact, previous studies have shown that asking sensitive questions about criminal behaviors yields more reliable estimates when measured through indirect questioning approaches [26–28]. Therefore, future studies in Iran and other conservative Islamic countries could consider employing indirect data collection methods to help provide a more accurate picture of alcohol consumption in their environments.

The findings of this study indicated that the frequency of AUD relapse is the best predictor of relapse. Secondly, a family history of physical illness was also a predictor for AUD relapse. Duka et al. [29] also showed that individuals with more previous attempts at alcohol detoxification had a higher rate of AUD relapse. Those who have experienced a higher number of attempts to quit alcohol are the same individuals who have relapsed more frequently, further supporting the findings of our study.

Regarding the high prevalence of AUD using indirect methods compared to direct methods, for implementing and developing specific AUD prevention programs, hidden populations in the society should be addressed. Also, using the experiences of other successful cities and countries in the field of reducing AUD, investigating the factors of people’s tendency to use alcohol, increasing anti-alcohol consumption policies, holding training programs, and informing people in society about increasing awareness about the complications as well as harms of alcohol use in the media, both directly and indirectly, more useful and effective steps can be taken to solve this problem.

This research faced some limitations. A potential limitation of this study is that all data on the prevalence of AUD were collected using a self-reported questionnaire without any biomedical markers. The main strength of this study was the use of a large sample size, which can represent the entire population of Kermanshah.

This study revealed the prevalence of AUD patterns in the population of Kermanshah Province and the factors associated with relapse. The results of this study can inform policymakers about the status of AUD. Additionally, these results help identify high-risk demographic groups across all age ranges, education levels, etc.

Although our general information about the prevalence of AUD is limited, this study provides a comprehensive view of the prevalence and severity of alcohol use in Kermanshah, which can aid in informing policies and planning efforts in public health and public policy sectors. By utilizing the findings of this study, factors that lead to substance relapse can be identified, and appropriate programs can be developed to reduce relapse. Furthermore, by identifying at-risk or affected populations, rehabilitation programs can be implemented to improve their conditions. In terms of research, the results of this study can be used for broader investigations, and in clinical practice, identifying individuals with influential characteristics in relapse or the onset of substance use disorders allows for the screening of individuals susceptible to these issues.

## Strengths and weaknesses

Since the present study was conducted in western Iran, generalizing the results of the study to other cultures and cities in Iran and the world should be done cautiously. The methods used to estimate the prevalence of AUD were not complete, though we used two indirect methods, NUS and CM here. In this study, we used a large sample size and trained individuals for data collection.

## Future directions

It is suggested that future research be performed longitudinally and on other provinces of Iran. Also, since the self-report tool was used in our study to estimate the prevalence of alcohol consumption, and this information may be subject to bias, it is suggested to use biological samples in future studies.

## Conclusion

The results of the present study showed that the estimated prevalence of AUD in Kermanshah Province, Iran, via indirect methods was higher than the direct prevalence. According to the findings, the rate of AUD in male significantly differed from that in female. Based on this research, the frequency of relapse is the best predictor of relapse history in individuals, with a family history of physical illness being the second predictive factor. Therefore, it is recommended that when planning AUD prevention policies, attention should be paid to hidden populations and socio-demographic factors in preventing AUD relapse.

## Supporting information

S1 File This study presents a comprehensive evaluation of the prevalence of Alcohol Use Disorder (AUD) and relapse risk factors in Kermanshah Province, Iran, using a combination of direct and indirect methods, including the Network Scale-Up (NUS) and Crosswise (CM) models, along with tree-based analysis. The findings reveal varying estimates of AUD prevalence and identify significant factors influencing relapse, such as family history of physical illness. These insights can guide healthcare interventions aimed at preventing AUD and enhancing relapse management in the region.
(SAV)
